# Macrophages promote a profibrotic phenotype in orbital fibroblasts through increased hyaluronic acid production and cell contractility

**DOI:** 10.1038/s41598-019-46075-1

**Published:** 2019-07-03

**Authors:** I-Hui Yang, Geoffrey E. Rose, Daniel G. Ezra, Maryse Bailly

**Affiliations:** 1 UCL Institute of Ophthalmology, London, EC1V 9EL UK; 2 Department of Adnexal Surgery, Moorfields Eye Hospital, London, EC1V 2PD UK; 3 NIHR Biomedical Research Centre for Ophthalmology, Moorfields Eye Hospital, City Road, London, EC1V 2PD UK; 4 Department of Ophthalmology, Kaohsiung Chang Gung Memorial Hospital and Chang Gung University College of Medicine, Kaohsiung, Taiwan

**Keywords:** Eye diseases, Experimental models of disease

## Abstract

Graves’ orbitopathy (GO) is an autoimmune inflammatory disease affecting the orbit. Orbital fibroblasts are a key component in GO pathogenesis, which includes inflammation, adipogenesis, hyaluronic acid (HA) secretion, and fibrosis. Macrophages are thought to participate in the immunological stage of GO, but whether they can directly affect the fibroblasts phenotype and modulate disease progression is unknown. We previously showed that GO adipogenic and fibrotic phenotypes could be modelled in a pseudo-physiological 3D environment *in vitro*. Here, we introduced macrophages in this 3D culture model to investigate role for macrophages in modulating adipogenesis, HA production, and contractility in orbital fibroblasts. Macrophages had a minimal effect on lipid droplet formation in fibroblasts, but significantly increased HA production and cell contractility, suggesting that they may promote the fibrotic phenotype. This effect was found to be mediated at least in part through phosphatidylinositol-4,5-bisphosphate 3-kinase (PI3K) activation and linked to an increase in actin polymerization and protrusive activity in fibroblasts. Overall our work shows for the first time a direct role for macrophages in modulating the fibroblasts’ phenotype in GO, supporting a role for macrophages in the progression of the fibrotic phenotype through induction of HA production and stimulation of the contractile phenotype in orbital fibroblasts.

## Introduction

Graves’ orbitopathy (GO) is an autoimmune inflammatory ophthalmopathy related to thyroid dysfunction. The impact of GO on patients varies from mild eye irritation to periocular disfigurement and severe visual impairment. GO is thought to stem from an initial autoimmune inflammatory reaction, followed by activation of local fibroblasts with resultant hyaluronic acid (HA) production and adipogenesis, and a later fibrotic stage. These pathological processes are responsible for a variety of clinical manifestations, including periocular erythema and swelling, protrusion of the eyeball, limitation of eye movements and double vision, and–in extreme cases–optic nerve compression and blindness^1^.

Ninety percent of GO occurs in Graves’ disease patients^2^. GO and Graves’ disease share a common immunopathogenesis, which is initiated by T cell intolerance to thyrotropin receptor (TSHR). Thyrocytes and orbital fibroblasts both express CD40 and TSHR as targets for CD40L(CD154) on T cells and TSHR auto-antibodies respectively, and thus make the thyroid gland and the orbit target organs for this autoimmune disease^1,3,4^. This autoimmunity has been found to be related to genetic susceptibility in human leukocyte antigen DR isotype (HLA-DR), CD40, thyroglobulin, and TSHR genes^5,6^. Histochemical studies in early GO show infiltrating orbital immunocompetent cells to be mainly T lymphocytes (predominantly CD4+) and macrophages, with a few B cells^7,8^. T cells can interact with orbital fibroblasts directly through CD40-CD40L bridges^3^, stimulating nuclear factor kappa-light-chain-enhancer of activated B cells (NF-κB) activation in orbital fibroblasts, and further enhancing inflammation through cytokine secretion^3,9^. The CD40-CD40L ligation also promotes fibroblast proliferation and HA synthesis and thus contributes to GO tissue remodelling^9,10^. The understanding of the role of macrophages in GO is, however, very limited.

A role for macrophages in the immunological stage of GO was initially evidenced by upregulation of macrophage-derived cytokine gene expression in GO tissues^11^. Along with other immune cells, monocytes and activated macrophages were found in active GO fibrovascular septae and orbital tissues by immunohistochemistry. As disease activity regresses, the number of activated macrophages decreases, but remains higher in fibrovascular septae than in adipose tissue when compared to respective areas in control tissues^12^. The expression of CD68, a macrophage-specific marker, was shown to be higher in GO tissues compared to control tissues, and correlated with GO severity^13–15^. Furthermore, in GO, macrophage infiltration is found around blood vessel walls^13^, among adipocytes^13,14^, and in fibrotic areas of adipose tissue^14^, suggesting that macrophages may be linked to the late fibrosis in GO fibrovascular tissues.

Macrophages have been shown to regulate remodelling and fibrosis of adipose tissue in obesity^16^. CD11c+ M1 macrophages, induced from monocytes, are involved in pro-inflammatory signalling, whereas resident CD11c− M2 macrophages contribute to the resolution of inflammation. M1 and M2 macrophages also act antagonistically in adipogenesis, whereby M1 inhibits adipogenesis whilst M2 promotes it^16,17^. Moreover, while M1 macrophages impair adipogenesis, they are thought to promote fibrosis by secretion of pro-inflammatory factors like transforming growth factor beta (TGF-β), which can drive myofibroblast differentiation through alpha-smooth muscle actin (αSMA) induction^18,19^. We therefore hypothesized that macrophages might play a similar dual role in GO, balancing pro-inflammatory, adipogenic and fibrotic stimuli to modulate disease phenotype. We used an *in vitro* 3D co-culture model^20,21^ to investigate the effect of macrophages on disease phenotype using primary orbital fibroblasts from control individuals or patients with GO. We demonstrate that while macrophages do not significantly promote adipogenesis, they stimulate HA production and a contractile phenotype in orbital fibroblasts, and thus may contribute to tissue remodelling and fibrosis, as well as perpetuating inflammation in the orbital tissue during GO progression.

## Results

### Macrophages have little effect on spontaneous lipid formation in 3D-cultured orbital fibroblasts

**Figure 1:**
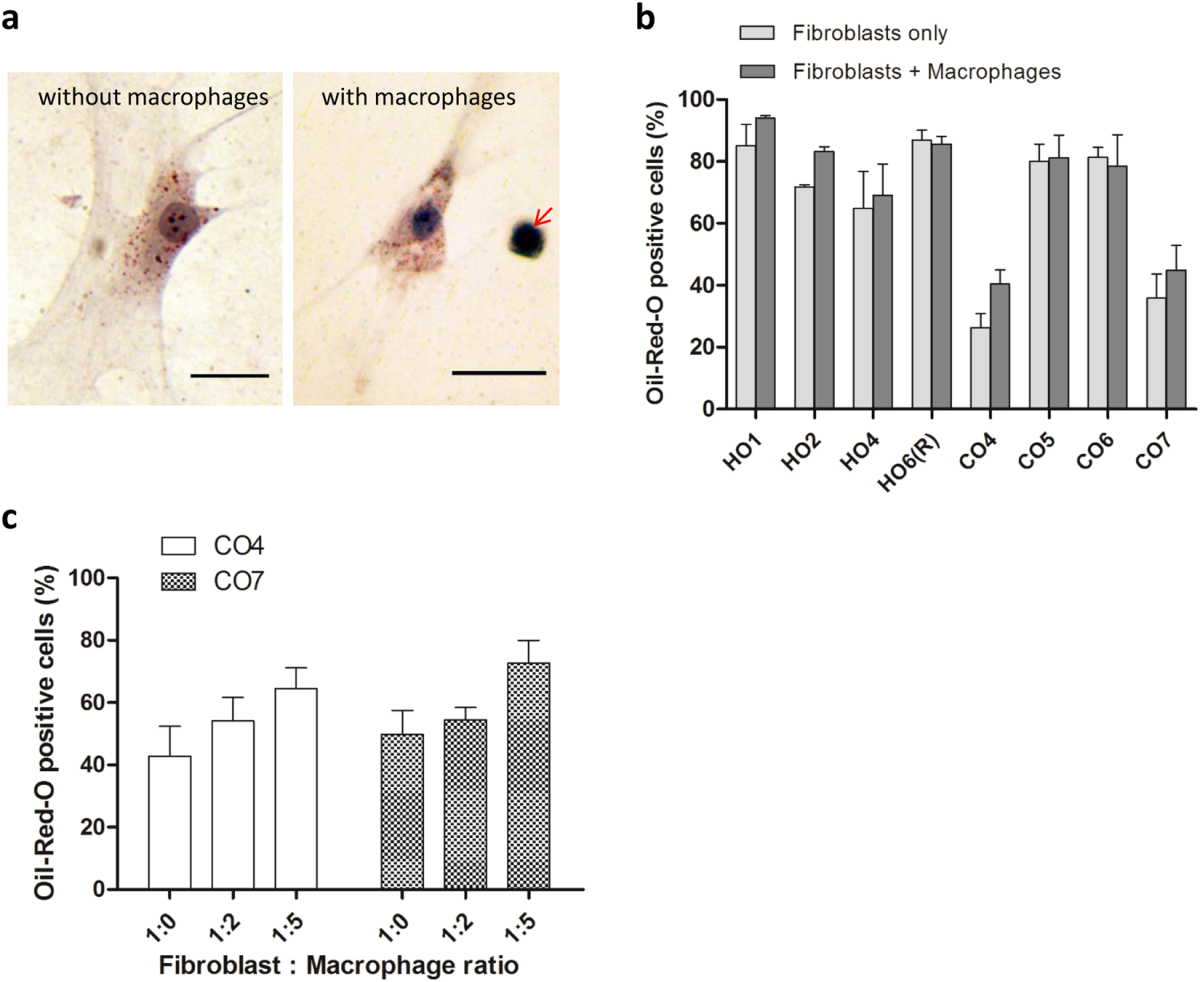
Macrophages do not significantly alter spontaneous lipid vesicle formation in 3D-cultured orbital fibroblasts. **(a)** Representative Oil-Red-O (ORO) staining of lipid-containing vesicles in orbital fibroblasts in 3D culture for 7 days: HO2 without macrophages (left) and CO6 with macrophages (right, arrow), at a 1:1 fibroblast:macrophage ratio. Scale bar, 25 μm. **(b)** Macrophages did not alter spontaneous lipid vesicle formation in 3D culture. GO (HOs) or control (COs) fibroblasts (0.74 × 10^5^ cells/ml) were cultured with macrophages at a 1:1 fibroblast:macrophage ratio in the presence of 10% serum for 7 days. The fraction of ORO positive cells is shown as mean ± SEM (n = 3, no statistical difference between with and without macrophages). **(c)** ORO positive cells in control fibroblast lines, CO4 and CO7, did not significantly increase as the number of macrophages increased (mean ± SEM; n = 4, *p* = 0.118 (CO4) and *p* = 0.123 (CO7), Kruskal-Wallis test).

As previously reported^20^, orbital fibroblasts in 3D culture in standard medium (DMEM with 10% serum) spontaneously produce lipid droplets, as evidenced by Oil-Red-O (ORO) staining (Fig. 1a *left*, 1b light grey bars). Co-culturing the fibroblasts with monocyte-derived human macrophages (U937 line) at a 1:1 ratio did not significantly alter their ability to produce lipid vesicles (Fig. 1a *right*, 1b dark bars), although there was a trend for an increase in ORO positivity in the two control orbital fibroblast lines with low levels of lipid vesicles. Further increasing macrophage proportion in the co-culture had only a moderate effect in lipid vesicle formation, with no significant increase even when macrophages were five times more abundant than fibroblasts (Fig. 1c). Overall this suggested that macrophages do not significantly modulate spontaneous lipid vesicle formation in orbital fibroblasts.

### Macrophages stimulate hyaluronic acid production in orbital fibroblasts

**Figure 2:**
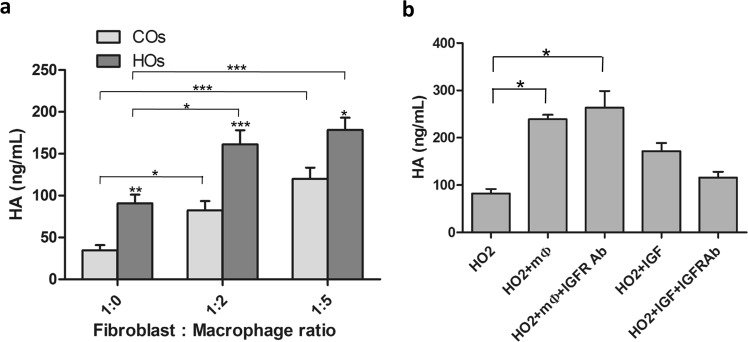
Macrophages stimulate hyaluronic acid (HA) production by orbital fibroblasts. **(a)** Control (COs) and GO-derived (HOs) orbital fibroblasts (0.74 × 10^5^ cells/ml) were cultured in collagen gels in serum-free medium with macrophages at different ratios, and HA levels were measured from pooled medium and gel digest after 3 days (mean ± SEM; COs: CO4, CO5, CO6, CO7; HOs: HO1, HO2, HO4, HO6(R); 3–4 independent experiments each). *p < 0.05, **p < 0.01, ***p < 0.001, Kruskal-Wallis test. Stars directly above HOs bars indicate the comparison between COs and HOs in that fibroblast:macrophage ratio group, Mann-Whitney test. **(b)** Macrophage-induced HA production is independent of IGF-1R signalling. HO2 cells were cultured with macrophages at 1:2 ratio or in the presence of 20 μM IGF-1, and IGF-1 signalling was blocked by adding IGF-1R blocking antibody 5 μg/ml in medium and 10 μg/ml in gel mix (n = 3, *p < 0.05, Kruskal-Wallis test).

Hyaluronic acid (HA) release in both 3D gels and medium was assessed using ELISA. Four control (“COs”: CO4, CO5, CO6, CO7) and four GO (“HOs”: HO1, HO2, HO4, HO6(R)) fibroblast lines were cultured without macrophages, or with macrophages at various ratios, in 3D collagen gels in serum-free medium (as high HA levels are present in serum; data not shown). In the absence of macrophages, GO orbital fibroblasts secreted significantly higher levels of HA compared to control orbital fibroblasts, consistent with previous reports using cells cultured as 2D monolayers^22^. Co-culturing orbital fibroblasts with macrophages at 1:2 and 1:5 ratio significantly increased HA production in both control and GO fibroblasts, with higher HA levels for GO orbital fibroblasts (Fig. 2a). Insulin-like growth factor-1 (IGF-1) working through insulin-like growth factor-1 receptor (IGF-1R) on orbital fibroblasts has classically been reported to activate HA secretion^22,23^. To determine whether macrophages stimulate HA production through IGF-1, a representative GO cell line, (HO2), was co-cultured with macrophages at 1:2 fibroblast:macrophage ratio in the presence of an IGF-1R blocking antibody. Inhibiting IGF-1R signalling did not affect macrophage-induced HA secretion although, as expected, it reduced IGF-1-mediated HA production in fibroblasts alone^22,24^ (Fig. 2b). This suggested that macrophages stimulate HA production in orbital fibroblasts independently of IGF-1R signalling.

### Macrophages promote orbital fibroblast contractility

**Figure 3:**
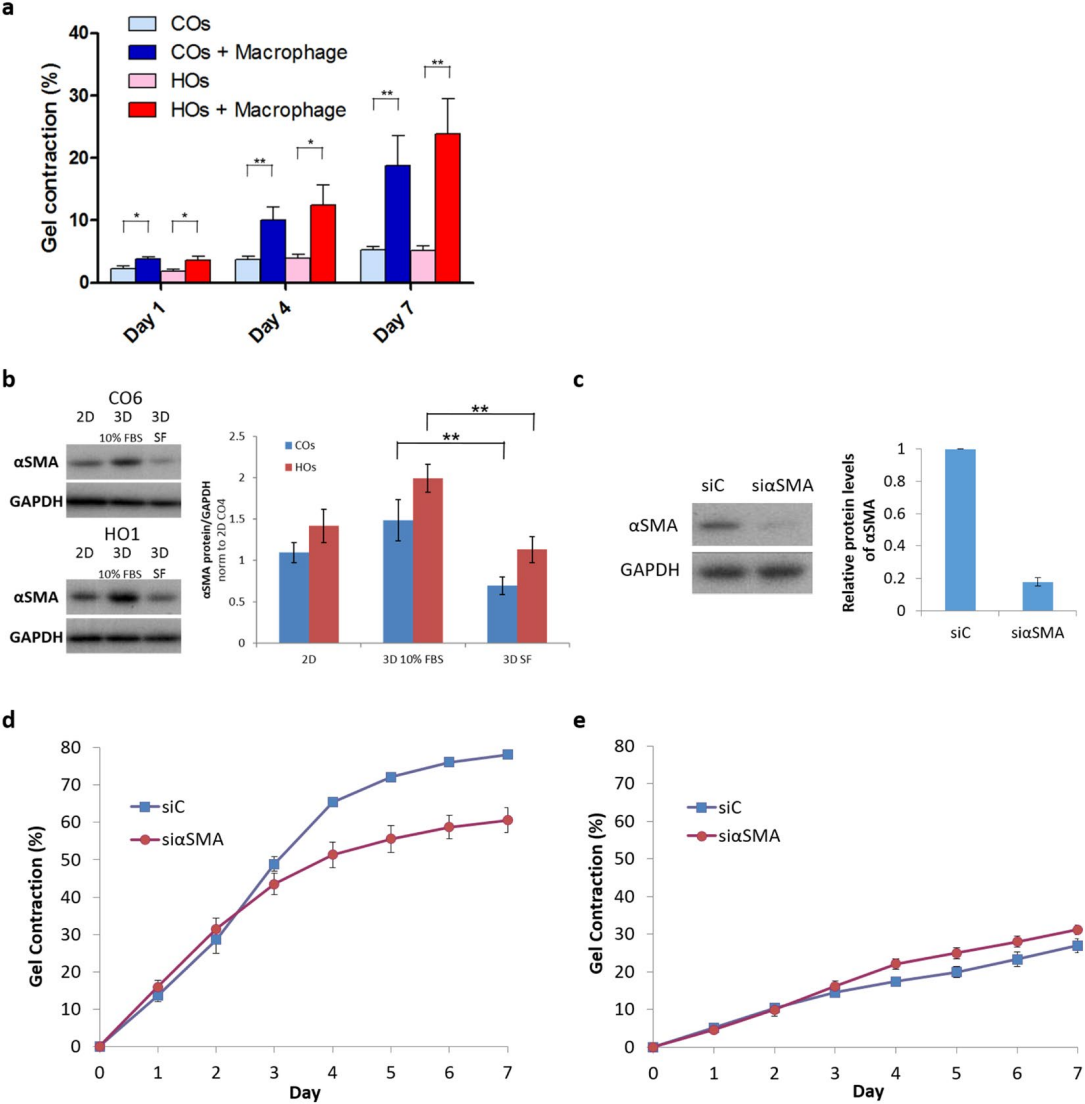
Macrophages promote orbital fibroblasts contractility independently from αSMA levels. **(a)** Orbital fibroblasts (1.48 × 10^5^ cells/ml) were cultured with macrophages (2.22 × 10^5^ cells/ml) in serum-free medium for 7 days. Gel contraction is shown as mean ± SEM. Control (COs) and GO (HOs) fibroblasts are each averaged from 3 lines (COs: CO4, CO6, CO7; HOs: HO1, HO2, HO4) with n = 3 and triplicates in each experiment. *p < 0.05, **p < 0.01, Mann-Whitney test. **(b)** Representative western blots and quantification of relative αSMA protein levels for the same 3 COs and 3 HOs cell lines after 5~7 days culture in the presence of 10% serum in monolayers (2D) and in gels (3D), and in serum-free medium in gels (SF) (n ≥ 3; **p < 0.01, Kruskal-Wallis test and post-hoc). Full-length blots are presented in Supplementary Fig. S1. **(c)** Representative western blot and quantification of αSMA levels showed effective αSMA knockdown by siRNA in HO2 (GO) orbital fibroblasts (n = 4). Full-length blots are presented in Supplementary Fig. S2. **(d)** Downregulating αSMA significantly decreased the contractility of HO2 fibroblasts in the presence of 10% serum (n = 3 and triplicates in each experiment, Day 7 *p* < 0.001, Mann-Whitney test). **(e)** Downregulating αSMA did not decrease macrophage-stimulated contraction of HO2 orbital fibroblasts in serum-free medium (n = 4 and triplicates in each experiment, Day 7 *p* = 0.215, Mann-Whitney test).

We previously showed that, in the presence of 10% serum, GO orbital fibroblasts contracted collagen matrix more efficiently than control fibroblasts and were more responsive to profibrotic and pro-inflammatory cytokines TGF-β1 and interleukin-1 beta (IL-1β)^20^. To determine whether macrophages alone could stimulate contraction of orbital fibroblasts, as they do for other ocular fibroblasts^21^, we co-cultured orbital fibroblasts in serum-free medium at a 2:3 fibroblast:macrophage ratio. Macrophages alone were unable to contract collagen gels^21^ (data not shown). Orbital fibroblasts were poorly contractile in serum-free medium^20^ and, therefore, higher numbers were needed to detect baseline contraction, these higher numbers of fibroblasts limiting the amount of macrophages that can be added to co-cultures without overcrowding the gels. Nevertheless, macrophages significantly promoted contraction for both control and GO fibroblasts (Fig. 3a).

We previously showed that orbital fibroblasts are highly contractile in the presence of serum, and responsive to TGF-β1^20^, a cytokine involved in promoting the myofibroblast fibrotic phenotype^25,26^. We thus sought to determine whether orbital fibroblast contractility was linked to expression of the classical fibrosis marker, αSMA. Both control and GO orbital fibroblasts expressed significant levels of αSMA when cultured in the presence of 10% serum, with αSMA expression being slightly higher in 3D as compared to 2D, and levels overall were consistently higher in GO cells. As expected, αSMA levels were significantly reduced in serum-free medium, with GO cells retaining more αSMA than control ones (Fig. 3b, full-length blots are presented in Supplementary Fig. S1). Downregulating αSMA to minimal levels using siRNA (Fig. 3c, full-length blots are presented in Supplementary Fig. S2) significantly reduced serum-mediated gel contraction in HO2 (GO line) cells (Fig. 3d), but did not affect macrophage-mediated contraction in the absence of serum (Fig. 3e). This suggested that macrophage-induced orbital fibroblasts contractility is distinct from serum-induced contractility, and not directly linked to αSMA levels.

### Macrophages stimulate actin polymerization and protrusion in orbital fibroblasts

**Figure 4:**
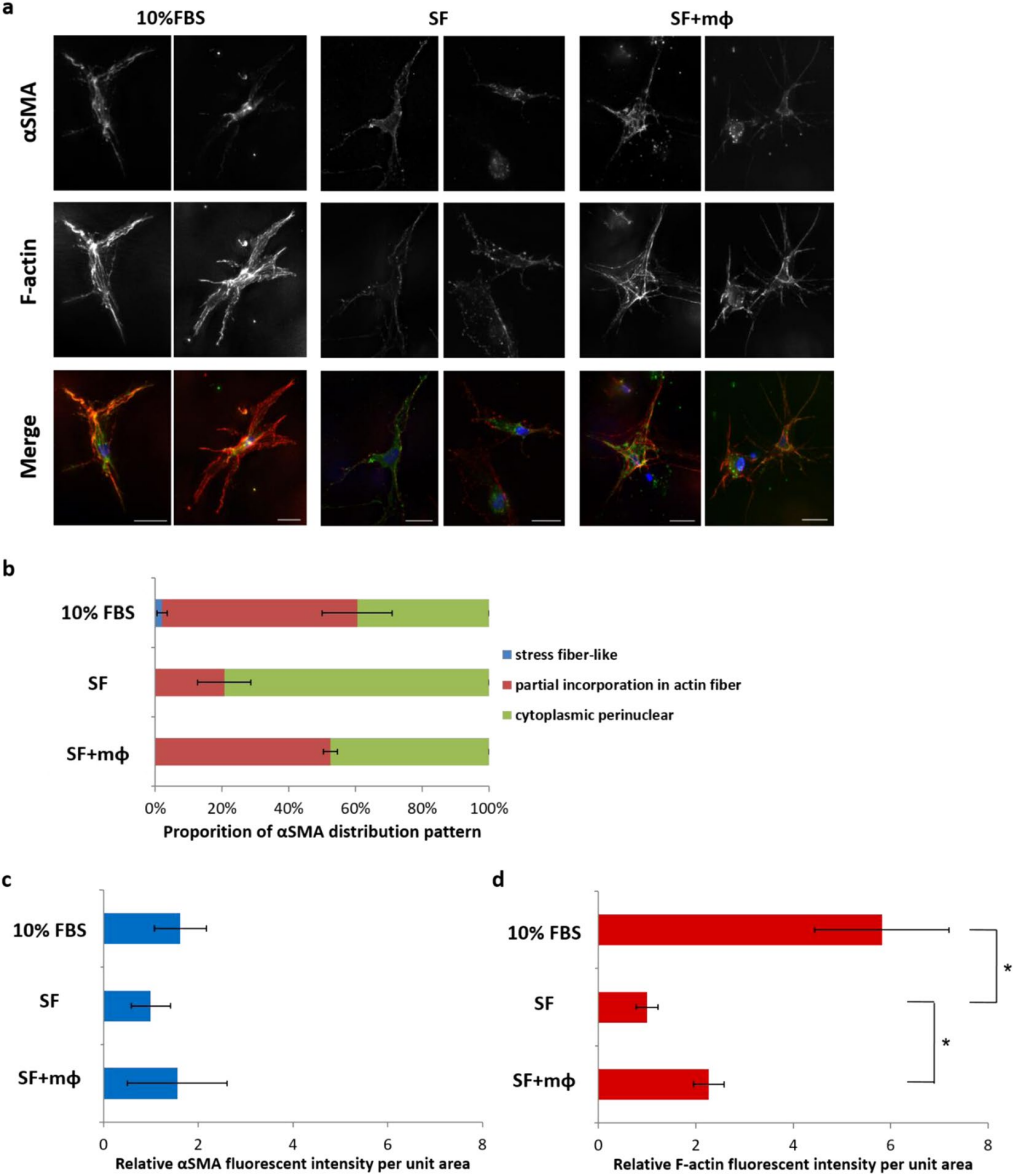
Macrophages increased F-actin levels and αSMA incorporation in actin fibers. **(a)** Representative images of αSMA and F-actin staining in HO2 fibroblasts following 4 days culture in gels in the presence of 10% serum (10%FBS), and in serum-free medium without (SF) and with macrophages (SF + mΦ). Scale bar, 25 µm. Merge images show αSMA (green), F-actin (red), and DAPI (blue). **(b)** αSMA distribution patterns in HO2 fibroblasts (>100 cells, n = 3). **(c**,**d)** αSMA (**c**) and F-actin (**d**) levels in HO2 cells in gels in the presence of 10% serum, and in serum-free medium with/without macrophages; shown are fluorescence intensity measurements from maximum intensity projections normalized to the value of serum-free condition (n = 4, *p < 0.05, Mann-Whitney test).

**Figure 5:**
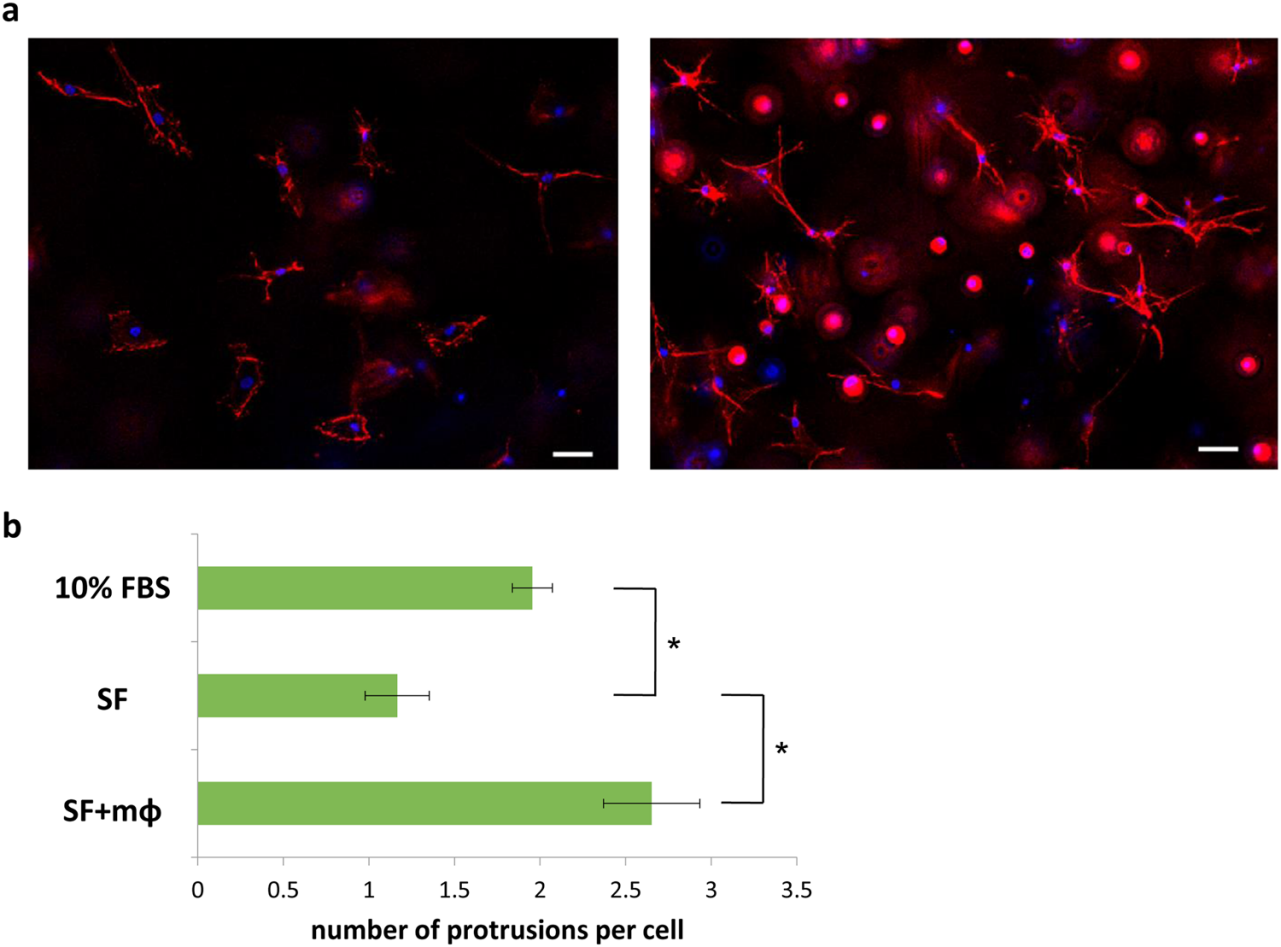
Macrophages stimulate protrusive activity of orbital fibroblasts. **(a)** Representative images of F-actin staining of HO2 cells in gels without (left) and with (right) macrophages in serum-free medium. Shown are maximal intensity projections of deconvolved image stacks. Scale bar, 50 µm. **(b)** Fibroblast protrusive activity with/without macrophage co-culture: shown is the average number of protrusions per cell, as determined using F-actin staining (n = 4, *p < 0.05, Mann-Whitney test).

To better understand how macrophages stimulate orbital fibroblast contraction, we examined fibroblast morphology in the gels during contraction. In the presence of serum, orbital fibroblasts adopted a “spread” morphology, with long, thick protrusions rich in filamentous and cortical actin, but lacking strong straight actin fiber bundles (Fig. 4a). Alpha-SMA staining partly overlapped with actin filaments, but only a minute proportion of the cells (less than 3%) displayed αSMA in a typical “stress fiber” pattern (Fig. 4a,b), suggesting that orbital fibroblasts do not differentiate into classical myofibroblasts^27–29^ during contraction in 3D gels. Rather, in the majority of cells, αSMA was either cytoplasmic (in a perinuclear pattern), or partially co-localized to F-actin patterns (cortical fibers or intracellular bundles; Fig. 4a,b). Under serum-free conditions, most cells displayed cytoplasmic perinuclear αSMA (Fig. 4b). Upon macrophage co-culture, there was no change in αSMA levels (Fig. 4a,c), but a significant increase in the proportion of cells with αSMA incorporation into actin bundles, with more than 50% of the cells showing partial co-localization with F-actin (Fig. 4b). This matched an increase in total F-actin observed in the cells in the presence of macrophages (Fig. 4d), suggesting that αSMA is randomly incorporated into actin fibers when they form as a result of the macrophage stimulation of actin polymerization. Accordingly, macrophage co-culture resulted in significant changes in fibroblast morphology: in serum-free medium, fibroblasts had few protrusions (Fig. 5a,b), matching low levels of polymerized actin (Fig. 4d), whilst fibroblasts adopted a more “stellate” appearance, with numerous long thin protrusions in the presence of macrophages (Fig. 5a,b). Increased levels of F-actin and protrusion numbers upon co-culture with macrophages suggest that macrophages directly stimulate actin polymerization and protrusive activity in fibroblasts, which presumably drives the resulting gel contraction.

### Macrophages promote the profibrotic phenotype through TGF-β and PI3K signalling

**Figure 6:**
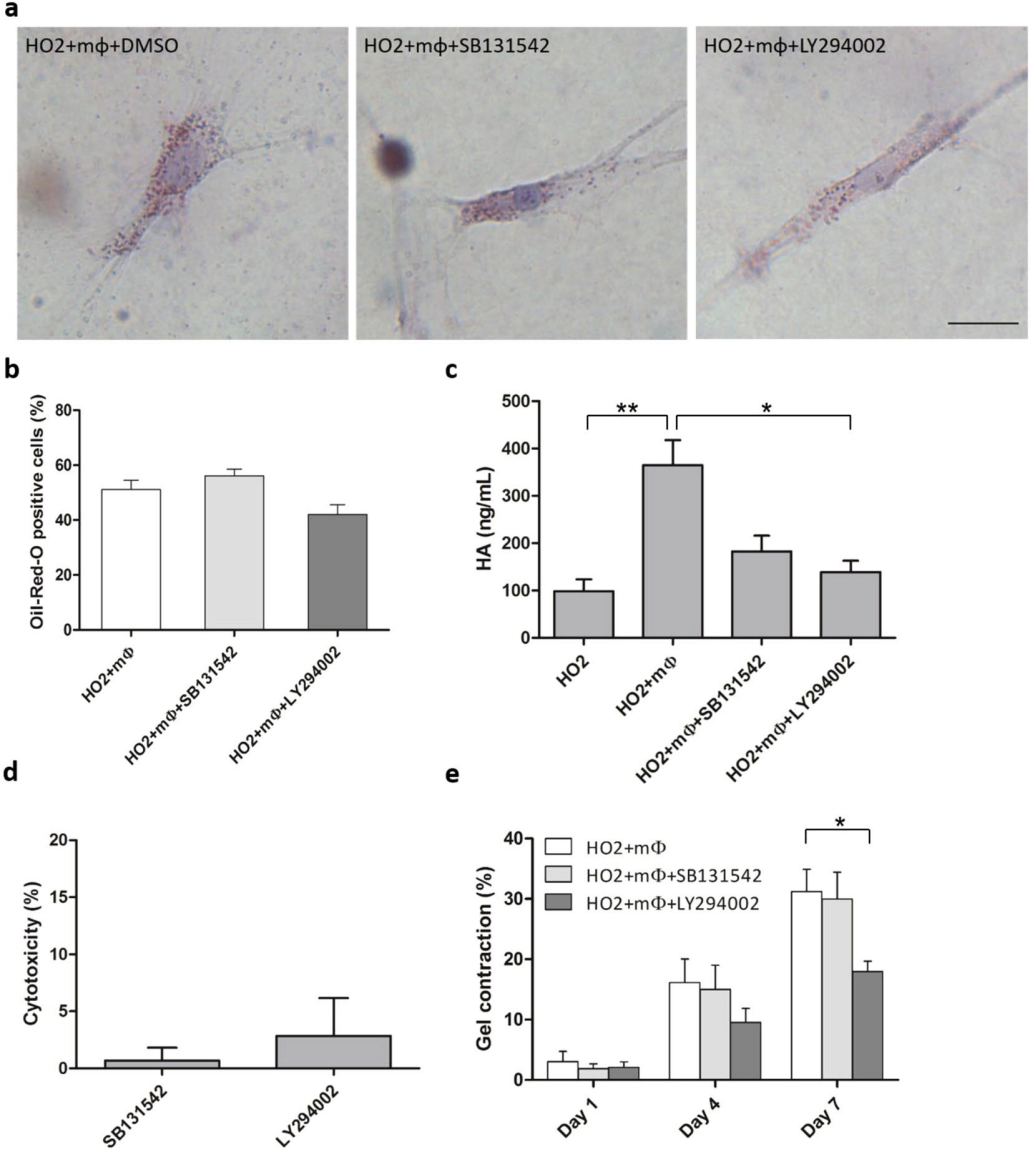
TGF-β and PI3K pathways differentially regulate macrophages’ effect on orbital fibroblasts fibrotic phenotype. **(a)** Representative images of ORO-stained HO2 fibroblasts (0.74 × 10^5^ cells/ml) co-cultured with macrophages (0.74 × 10^5^ cells/ml) in 3D for 7 days in the presence of DMSO control (0.1% DMSO), TGF-β (SB431542, 10 μM) and PI3K (LY294002, 10 μM) inhibitors. (Scale bar, 25 μm). **(b)** TGF-β (SB431542, 10 μM) and PI3K (LY294002, 10 μM) inhibitors did not alter lipid vesicle formation in HO2 cells co-cultured with macrophages. HO2 fibroblasts (0.74 × 10^5^ cells/ml) were cultured in gels with macrophages (0.74 × 10^5^ cells/ml) for 7 days and the proportion of cells containing lipid vesicles was counted after Oil-Red-O staining (Mean ± SEM, n = 4, *p* = 0.343 (SB131542) and *p* = 0.2 (LY294002), Mann-Whitney test). **(c)** TGF-β and PI3K inhibition prevented macrophage-mediated HA production. HO2 fibroblasts (0.74 × 10^5^ cells/ml) were co-cultured with macrophages at 1:2 fibroblast:macrophage ratio in gels with/without inhibitors and HA levels were measured after 3 days (n = 3, *p < 0.05, **p < 0.01, Kruskal-Wallis test and post-hoc). **(d)** LDH assay showed minimal cytotoxicity of SB131542 and LY294002 after 3 days in 1:2 HO2:macrophage co-culture 3D gels. Shown is calculated toxicity normalized to 100% toxicity control (n = 2). **(e)** HO2 cells (1.48 × 10^5^ cells/ml) were co-cultured with macrophages (2.22 × 10^5^ cells/mL) in collagen gels in serum-free medium for 7 days for contraction assay, with/without TGF-β or PI3K inhibitors. (Mean ± SEM, n = 4, *p < 0.05, Mann-Whitney test).

TGF-β signalling is reportedly involved in obesity^30^, HA production^31^ and fibrosis in various organs^32^. Similarly, phosphatidylinositol-4,5-bisphosphate 3-kinase (PI3K) has been implicated in lipid accumulation^33^, and the PI3K-Akt pathway was previously shown to regulate HA production in GO fibroblasts^34^. Furthermore, whether as an independent pathway or through non-canonical TGF-β signalling, PI3K is a frequent target for anti-fibrotic therapies^35^. Activated macrophages can produce TGF-β and other pro-inflammatory cytokines that may activate these pathways in fibroblasts and promote fibrosis^36^. To determine whether either pathway could be modulating macrophage-mediated stimulation of orbital fibroblasts, we assessed the effect of TGF-β (SB431542, 10 μM) and PI3K (LY294002, 10 μM) inhibitors on lipid vesicle formation, HA production and gel contraction using HO2 (GO line) cells in the presence of macrophages. Neither inhibitor affected spontaneous lipid vesicle formation (Fig. 6a,b), but both SB431542 and LY294002 decreased macrophage-induced HA production (Fig. 6c), with minimal cytotoxicity (Fig. 6d). However, only PI3K inhibition significantly decreased macrophage-induced gel contraction (Fig. 6e).

## Discussion

GO is an autoimmune inflammatory disease, associated in its active phase with both T and B cell inflammatory reactions^8,15^. TSHR is thought to be the key auto-antigen in GO, as it is expressed in orbital fibroblasts and acts in cooperation with IGF-1R to drive the phenotypic changes for GO, such as adipogenesis, HA secretion, and fibrosis. Both circulating TSHR auto-antibodies and T cell-mediated inflammatory reactions have been implicated in GO^3,8,10^, and TSHR/IGF-1R activation has been linked to HA production^23,24,37^ and adipogenesis^38,39^. The role of B cells in the pathogenesis of GO and B-cell targeting anti-CD20 antibodies^40,41^ remains controversial, with conflicting data from recent clinical trials. The exact disease mechanism for GO has, however, remained elusive and what drives progression to the disfiguring fibrotic stage is still largely unknown.

We have used our *in vitro* model of GO to explore the possible role of a third inflammatory component, macrophages, in modulating the phenotype of GO orbital fibroblasts. Along with other inflammatory cells, monocytes and activated macrophages are found in orbital tissues, with their numbers significantly increased in active GO^12–14,42^. Tissue macrophages in GO are seemingly derived from circulating monocytes, rather than resident macrophages^12^, possibly representing a pro-inflammatory and profibrotic M1 population as described in obesity^16^. These activated macrophages are found around blood vessels and mature adipocytes in GO, and it is thought that they may stimulate disease progression through secretion of a range of cytokines, whose expression have been found elevated in GO tissues^12,13,42,43^. However no study yet has looked at a direct effect of macrophages on the orbital fibroblasts’ phenotype. We have shown previously that U937-derived macrophages are a good model for tissue-activated monocytes, secreting a large range of cytokines and able to activate both normal, and more specifically fibrotic, ocular fibroblasts^21^. U937-derived macrophages can promote a pro-inflammatory and profibrotic phenotype in conjunctival fibroblasts, presumably mimicking activated circulating macrophages^21^. Consistent with this, we show here that they also promote classical fibrotic features in orbital fibroblasts (such as HA secretion and increased contractility), with a more pronounced effect on GO fibroblasts. Interestingly, macrophages did not significantly affect spontaneous adipogenesis, consistent with our prior observation that the adipogenic and fibrotic phenotype in orbital fibroblasts are triggered through separate pathways^20^. Additionally, their lack of effect on the adipogenic phenotype suggest that they may be more similar to pro-inflammatory/profibrotic M1 macrophages^16^, consistent with histopathological evidence from GO tissues^12^.

Our work shows for the first time that macrophages can trigger HA secretion in orbital fibroblasts in a pseudo-physiological environment, especially in GO orbital fibroblasts. HA levels in turn may regulate fibroblast proliferation, thereby contributing to fibrosis^44^. Macrophages’ stimulation of HA production is likely linked to the cytokines and growth factors they produce. Indeed, macrophages secrete IL-1β^45^ and PDGF, which were shown to induce hyaluronan synthase 2 (HAS2) gene expression and HA production in orbital fibroblasts from GO patients^42,46^. Interestingly, macrophage activity may also be further activated by fibroblast-released HA, either through direct stimulation of HA receptor CD44 on the macrophage^47^ or by co-stimulation with chemokines^48^, thus creating a positive feedback loop that may sustain and exacerbate fibrosis. Prior studies have linked IGF-1R stimulation to HA production in orbital fibroblasts^22,34^, possibly through PI3K-A signalling^34^. Consistent with this, macrophage-mediated HA secretion was prevented by PI3K inhibitor LY294002. However, blocking IGF-1R did not prevent HA secretion following macrophage stimulation, suggesting that fibroblasts may use a different upstream activator of PI3K-A signalling to trigger HA production and secretion. Interestingly, activated macrophages are known to secrete TGF-β1^36^, and macrophage-mediated HA production was partially inhibited by blocking TGF-β receptor in HO2 (GO) cells in our model. A cross-talk between TGFβ1-TGFβ1 receptor/ALK5 and PI3K has been reported in a number of cell processes^49,50^, and it is thus possible that macrophages stimulate HA production in orbital fibroblasts through a non-canonical TGF-β1/PI3K pathway^35,51^.

An increase in fibroblast contractility is a hallmark of fibrosis, and we have shown previously that fibrotic ocular fibroblasts, including GO orbital fibroblasts, display increased contractile features in our 3D model compared to their normal, non-diseased counterparts^20,21,52^. Contractile fibrotic fibroblasts *in vivo* often present with characteristic cytoskeletal features, including expression of the specific actin isoform αSMA, defining the so-called myofibroblast phenotype^53^. *In vitro*, αSMA incorporation into strong actin bundles and prominent stress fibers on stiff or “tensioned” substrates have traditionally been linked to fibroblast contractility and myofibroblast phenotype^27^. As orbital fibroblasts displayed cytoskeletal changes, including an increase in actin bundles, after co-culture with macrophages, we initially hypothesized that macrophage-induced orbital fibroblasts contractility may be mediated through the acquisition of a myofibroblast-like phenotype. However, while both control and GO orbital fibroblasts express αSMA, we found that macrophage-mediated stimulation of fibroblast contractility was not linked to αSMA expression; rather, macrophages stimulated actin dynamics in fibroblasts, with increased protrusive activity and actin polymerization. We have shown previously that such dynamic protrusive activity in 3D gels is sufficient to generate contractile force in primary human fibroblasts, and may represent an alternate pathway to the fibrotic phenotype^21,54–56^. Consistent with this, macrophage-mediated contractility in orbital fibroblasts was not prevented by blocking TGF-β signalling, the classical upstream pathway mediating the acquisition of the myofibroblast phenotype^57^. Rather, PI3K may be mediating fibroblast contractility in the presence of macrophages, although we cannot discount the possibility that the PI3K inhibitor directly acted on the macrophages and prevented them from activating the fibroblasts, irrespective of the pathway used by the fibroblasts for contractility.

A body of work over the last decade has emphasized the importance of the mechanical environment in regulating cell phenotype in physiology and disease. Our 3D culture model of orbital fibroblasts mimics the low stiffness conditions encountered in the orbital fat tissue, and allows for the cells to express their intrinsic phenotype^20,58^. We show here that it can also be used to investigate interactions with immune cells and how they may regulate the fibroblasts phenotype, providing a valid model to dissect disease mechanism in human cells. Although the present study is limited by the relatively small number of fibroblast lines studied and the use of a cell line to generate macrophages, it identifies new potential mechanisms for disease progression. With the recent developments in animal models, this provides an ideal set up to further the understanding of the cellular biology of GO.

In conclusion, we show that macrophages stimulate HA production and contractile behaviour in orbital fibroblasts, suggesting they may play a key role in the fibrotic stage of GO. Further work will be needed to determine exactly how macrophages interact with fibroblasts, and at which stage of the disease their role might be critical. Interestingly, while signalling through PI3K appears to be one of the main drivers of the fibrotic phenotype, macrophages are able to trigger both HA production and contractility through non-canonical pathways, suggesting a potential for novel approaches to future treatment.

## Materials and Methods

### Cell culture

Fibroblasts were obtained from orbital adipose tissue through primary culture. Patient enrolment was approved by the National Health Service Research Ethics Service (NRES) Committee for East Midland (Leicester- REC reference number 14/EM/1186). Informed consent was obtained for all donors and all experiments were performed in accordance with local ethics guidelines and regulations. Orbital fat was harvested from patients with active GO undergoing orbital decompression surgery or from control individuals undergoing orbital surgery unrelated to GO^20^ (Table S1, Supplementary Information). Adipose tissues were chopped into 1~2 mm^2^ fragments and maintained in Dulbecco’s modified Eagle’s medium (DMEM; 4.5 g/L glucose; GIBCO, Thermo Fisher Scientific, UK) supplemented with Foetal Bovine Serum (FBS, 10% vol/vol; Sigma-Aldrich, UK), penicillin (100 U/ml), streptomycin (100 μg/ml), and L-glutamine (2 mM; all GIBCO, Thermo Fisher Scientific, UK) and incubated at 37 °C and 5% CO_2_. After cells grew out of the tissue fragments and reached confluence, they were trypsinized and maintained in the above medium. For all experiments, the resulting GO (HO) and control (CO) primary orbital fibroblasts lines were used between passages 3 and 9.

Macrophages were derived by phorbol-12-myristate-13-acetate (PMA; Sigma-Aldrich, UK) activation of U937 monocytes. Human monocyte cell line U937 was kept in RPMI 1640 medium (Sigma-Aldrich, UK) supplemented with 10% FBS, penicillin (100 U/ml), streptomycin (100 μg/ml), and L-glutamine (2 mM). For differentiation, 2 × 10^6^ cells were stimulated with PMA (150 nM) in medium for 72 hours. The medium was then replaced with fresh RPMI without PMA and the cells left for another 48~72 hours to complete differentiation into macrophages^21^.

### Three-dimensional (3D) cultures

A 3D collagen gel culture environment was established by embedding cells in 1.5 mg/ml collagen type 1 matrix (First Link Ltd., Birmingham, UK), as previously described^20,21^, using 0.74 × 10^5^ cells/ml orbital fibroblasts alone or with macrophages at 1:2 (1.5 × 10^5^ cells/ml) or 1:5 (3.7 × 10^5^ cells/ml) ratios. For gel contraction assays and immunofluorescence experiments, 150 μl gels were cast in the microwells of glass bottom MatTek dishes (MatTek Corporation, MA, USA), and then detached as free-floating gels^20^. For all other experiments, gels were cast in 24-well plates with 210 μl gel solution and left attached. Where appropriate, inhibitors (IGF-1R blocking antibody 1H7, 5 μg/ml, BioLegend, CA, USA; TGF-β inhibitor SB431542, 10 μM, Cayman Chemical, MI, USA; PI3K inhibitor LY294002, 10 μM, Cell Signaling Technology, MA, USA) or control diluent at matching concentration were added to the culture medium immediately after gel polymerization and maintained throughout the experiment (IGF-1R blocking antibody 1H7, 10 μg/ml was also added to the gel mix).

### Oil-Red-O staining

After 7 days of culture, 3D gels were rinsed with PBS and fixed in 10% formaldehyde for 20 minutes. Gels were then detached from the bottom of wells and washed in distilled water. After 5 minutes of incubation in 60% isopropanol, gels were stained for 2 minutes with freshly filtered Oil-Red-O (ORO) [working solution made of 3:2 vol/vol dilution of ORO stock (3 mg/ml in 99% isopropanol) and distilled water]. The gels were then rinsed in water and counterstained with hematoxylin (Gill No. 3, Sigma-Aldrich, UK) for 30 seconds, followed by several long washes in tap water. Gels were topped with a 50% glycerol in Tris-buffered saline (TBS) solution before observing and imaging. The ORO staining was quantified by counting ORO positive cells directly under the microscope (Leica DMIL, 20x objective). A cell was deemed “ORO positive” when harbouring 10 or more lipid droplets in its cytoplasm. A minimum of 100 cells were evaluated from 4~5 fields in each gel.

### Hyaluronic acid enzyme-linked immunosorbent assay (ELISA)

Hyaluronic acid (HA) production was quantified by ELISA following manufacturer’s guidance (R&D Systems, MN, USA). Fibroblasts, with or without macrophages, were cultured in collagen gels in serum-free medium with or without 5 μg/ml IGF-1R blocking antibody 1H7. After 3 days, the culture medium was collected and the gels digested with 0.05% collagenase-D (Roche, UK) for 30 min at 37 °C to, extract any further HA trapped in the gel. The collagenase digestion was centrifuged to remove cells and debris and the supernatant collected. The mixture of media and supernatant from digested gels was diluted in serum-free DMEM (1:30), and assayed according to the manufacturer’s instructions. Gels without cells, and gels with macrophages only, were used for baseline calibration. To confirm the efficiency of the blocking antibody, HA levels were measured in fibroblasts cultured in serum-free medium with 20 μM IGF-1 (R&D Systems, MN, USA) to stimulate HA production, with or without IGF-1R blocking antibody.

### Gel contraction assay

Gels were made in MatTek dishes for contraction assay as described^20^. Orbital fibroblasts at a concentration of 1.5 × 10^5^ cells/ml and macrophages of 2.25 × 10^5^ cells/ml were used in co-culture experiments. The gels were photographed daily (Nikon Coolpix camera) for 7 days, and gel area was measured using ImageJ (https://imagej.nih.gov/ij/). The contraction was expressed as the percentage of gel area decrease compared to the original gel area.

### Western blotting

Cells in 2D monolayer culture were trypsinized, pelleted and resuspended in RIPA lysis buffer [150 mM NaCl, 0.1% Triton X-100, 0.1% sodium dodecyl sulphate, 50 mM Tris-HCl pH 8.0, 0.5% sodium deoxycholate, protease inhibitors]. Cells in 3D collagen gels were obtained by digesting the gels with 0.05% collagenase-D until completely liquefied. The digest was centrifuged and the cell pellet resuspended in RIPA buffer. Protein concentration was quantified by BCA assay (Thermo Fisher Scientific, UK), and the samples were denatured in sample buffer (Thermo Fisher Scientific, UK) at 95 °C for 5 minutes. 7 μg of protein were loaded on 4–12% precast polyacrylamide gels (Novex™ Wedgewell™ or Bolt™, Invitrogen, Thermo Fisher Scientific, UK) for SDS-PAGE. Proteins were transferred to PVDF membranes (Thermo Fisher Scientific, UK), and then membranes were blocked for 1 hour at room temperature in 5% milk in 0.1% Tween-20 in TBS (TBS-T) before overnight incubation with primary antibody (anti-αSMA, 1:3000, A2547, Sigma-Aldrich, UK) at 4 °C. Membranes were washed with TBS-T before 1 hour of incubation with secondary antibody (Peroxidase-AffiniPure goat anti-mouse IgG (H + L), 1:5000, Jackson ImmunoResearch, UK) diluted in 5% milk in 0.1% TBS-T at room temperature. After another wash with TBS-T, membranes were developed using ECL (Pierce, Thermo Fisher Scientific, UK). For loading control, membranes were then washed, stripped with stripping buffer (Thermo Fisher Scientific, UK), blocked with 5% milk, and then re-probed with rabbit anti-GAPDH antibody (1:3000, ab9485, abcam, UK) in 5% milk and secondary antibody (Peroxidase-AffiniPure goat anti-rabbit IgG (H + L), 1:5000, Jackson ImmunoResearch, UK). Films were developed, scanned and analyzed using ImageJ software. Band intensities were normalized to GAPDH and then normalized to a reference sample loaded in each gel for comparing all cell lines and independent experimental repeats.

### Gene silencing

Gene silencing experiments were performed using siRNAs targeting ACTA2 (gene encoding αSMA, siGENOME SMARTpool M-003450-00-00005, Dharmacon, CO, USA) and non-target control (AllStars Negative Control siRNA, Qiagen). Fibroblasts were plated in 60 mm dishes (1.2 × 10^5^ cells/dish), and treated with 5 nM siRNA together with 3 μl/ml Hiperfect transfection reagent (Qiagen) for 3 days according to manufacturer’s instructions. Protein depletion was confirmed by Western blotting.

### Immunofluorescence

Orbital fibroblasts (1.5 × 10^5^ cells/ml) without or with macrophages (2.25 × 10^5^ cells/ml) were cultured in free-floating gels for 4 days, and processed for immunofluorescence using Rhodamine-labeled Phalloidin (Molecular Probes, OR, USA), anti-αSMA primary antibody (Sigma-Aldrich, UK) and Alexa Fluor 488-Conjugated AffiniPure donkey anti-mouse IgG (Jackson Immunoresearch, UK) as previously described^59^. Gels were mounted with Fluoroshield mounting medium with DAPI (abcam, UK), and imaged using a Nikon Eclipse Ti microscope with 10X Plan Fluor 0.3NA or 40X S Plan Fluor ELWD 0.60NA objectives. For quantification, full stacks (50 μm in Z stack with 1 μm step) of 5–6 fields were acquired using the 10X objective and processed using the NIKON NIS Element Software. Maximal intensity projections were generated and cells manually traced to get outlines as selected areas for calculations. Background signal was subtracted before calculating fluorescence intensity under TRITC or FITC channels. The fluorescence intensities of TRITC and FITC were then divided by the selected area to obtain fluorescence intensity per unit area. For representative single cell images, cells were imaged with 40x objective, processed by deconvolution, and shown as maximal intensity projections.

### LDH cytotoxicity assay

3D collagen gels were made with orbital fibroblasts (0.74 × 10^5^ cells/ml) and macrophages (1.5 × 10^5^ cells/ml) with/without TGF-β inhibitor SB431542 or PI3K inhibitor LY294002. After 3 days of incubation in phenol red-free and serum-free medium, culture medium was transferred to 96-well plate and processed using Pierce LDH cytotoxicity assay kit (Thermo Fisher Scientific, UK) and cytotoxicity calculated following manufacturer’s instructions. Cells lysed with lysis buffer were used as maximum LDH activity controls.

### Statistics

Statistical analysis was performed using two-tailed Mann-Whitney test or Kruskal-Wallis test with post-hoc Dunn’s multiple comparison test on Prism 5 (GraphPad software). *P* < 0.05 was considered statistically significant.

## Supplementary information

Dataset_1
