# Supplementary material for: Macrophages promote a profibrotic phenotype in orbital fibroblasts through increased hyaluronic acid production and cell contractility
Source: Sci Rep. 2019 Jul 3;9:9622. doi: 10.1038/s41598-019-46075-1 (PMC6610127; doi:10.1038/s41598-019-46075-1)
Supplement: Supplementary file 1 — Dataset_1 [file 41598_2019_46075_MOESM1_ESM.pdf]

Supplementary Information

Macrophages promote a profibrotic phenotype in orbital fibroblasts through increased hyaluronic acid production and cell contractility

I-Hui Yang [1,4], Geoffrey E. Rose[2], Daniel G. Ezra[1,2,3], and Maryse Bailly[1*]

[1] UCL Institute of Ophthalmology, London EC1V 9EL, UK.

[2] Department of Adnexal Surgery, Moorfields Eye Hospital, London EC1V 2PD, UK.

[3] NIHR Biomedical Research Centre for Ophthalmology, Moorfields Eye Hospital, City Road, London EC1V 2PD, UK.

[4] Department of Ophthalmology, Kaohsiung Chang Gung Memorial Hospital and Chang Gung University College of Medicine, Kaohsiung, Taiwan.

*** Correspondence:** m.bailly@ucl.ac.uk

Cell strain	Age	Gender	Surgical procedure	GO duration (months)	CAS score
HO1	71	F	Orbital decompression	15	6
HO2	60	M	Orbital decompression	8	5
HO4	49	F	Orbital decompression	7	6
HO6	58	F	Orbital decompression	9	5
CO4	49	M	Evisceration	n/a	n/a
CO5	80	M	Canthoplasty	n/a	n/a
CO6	56	F	Fat prolapse excision	n/a	n/a
CO7	33	M	Lacrimal gland exploration	n/a	n/a

Supplementary Table S1. Demographic data of tissue source for orbital fibroblasts. HOs were derived from patients with active GO undergoing orbital decompression surgery; COs were from control individuals undergoing orbital surgery unrelated to GO.

Figure S1

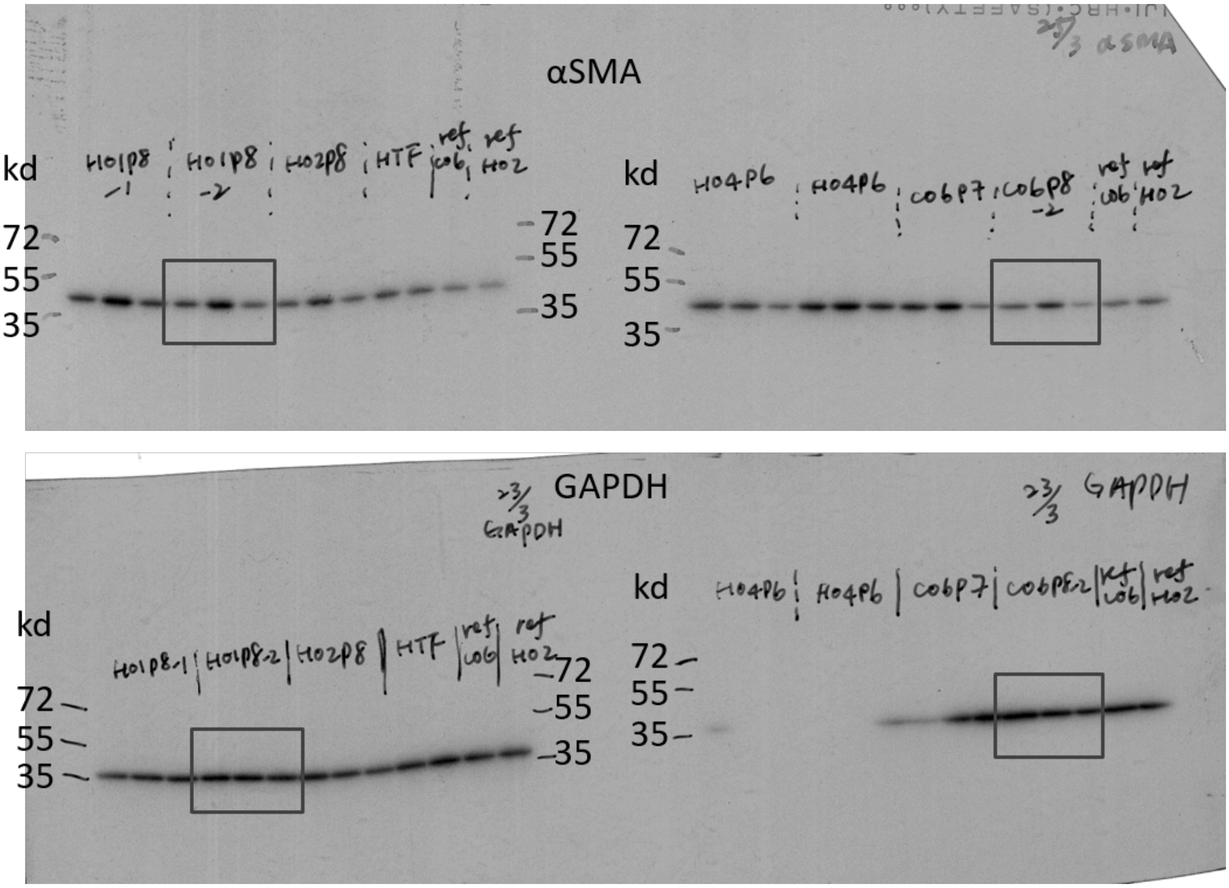

Supplementary Figure S1. Full-length Western blots of Figure 3B. Top panel was probed with αSMA, and bottom panel was probed with GAPDH. For semi-quantitative analysis, αSMA signals were normalized with GAPDH signals. Frames are the areas selected for representative images in Figure 3B. A loading control (ref HO2) was used for quantitative comparison between different blots.

Figure S2

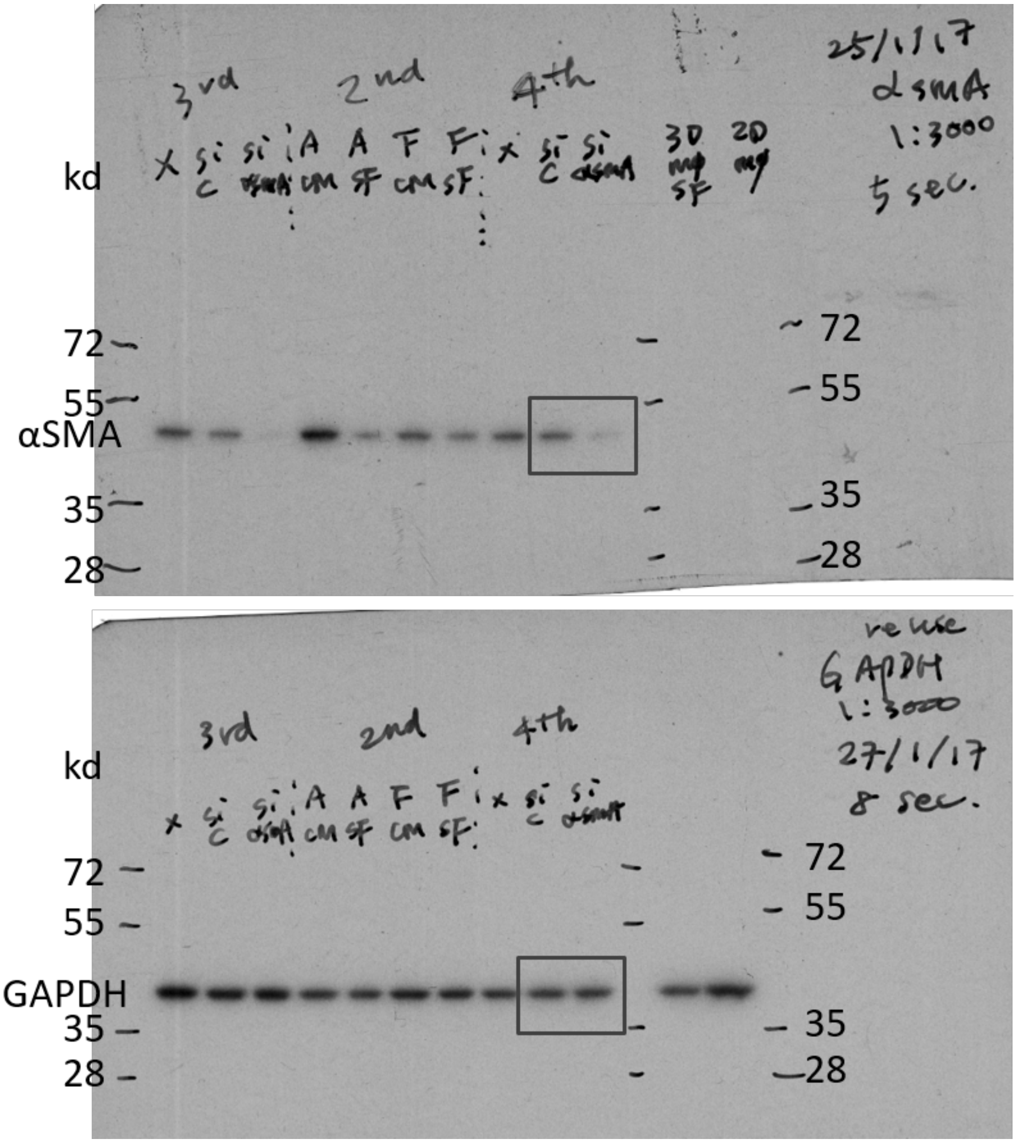

Supplementary Figure S2. Full-length Western blots of Figure 3C. Top panel was probed with αSMA, and bottom panel was probed with GAPDH. For semi-quantitative analysis, αSMA signals were normalized with GAPDH signals. Frames are the areas selected for representative images in Figure 3C.
